# Multiple functions of the ALT favorite helicase, BLM

**DOI:** 10.1186/s13578-025-01372-3

**Published:** 2025-03-01

**Authors:** Shun Chang, Jiang Tan, Ren Bao, Yanduo Zhang, Jinkai Tong, Tongxin Jia, Jing Liu, Juhua Dan, Shuting Jia

**Affiliations:** 1 Laboratory of Molecular Genetics of Aging and Tumor, Medical School, Kunming University of Science and Technology, 727 Jing Ming Nan Road, Kunming, Yunnan 650500 China; 2 Department of Neurosurgery, The First People’s Hospital of Yunnan Province, The Affiliated Hospital of Kunming University of Science and Technology, No.157 Jinbi Road, Kunming, Yunnan 650032 China

**Keywords:** BLM, Alternative lengthening of telomeres (ALT), Telomere, Replication stress

## Abstract

Eukaryotic somatic cells undergo continuous telomere shortening because of end-replication problems. Approximately 10%~15% of human cancers rely on alternative lengthening of telomeres (ALT) to overcome telomere shortening. ALT cells are characterized by persistent telomere DNA replication stress and rely on recombination-based DNA repair pathways for telomere elongation. The Bloom syndrome (BLM) helicase is a member of the RecQ family, which has been implicated as a key regulator of the ALT mechanism as it is required for either telomere length maintenance or telomere clustering in ALT-associated promyelocytic leukemia bodies (APBs). Here, we summarize recent evidence detailing the role of BLM in the activation and maintenance of ALT. We propose that the role of BLM-dependent recombination and its interacting proteins remains a crucial question for future research in dissecting the molecular mechanisms of ALT.

## Background

Telomeres are nucleoprotein complexes that protect the ends of linear chromosomes from being recognized as DNA damage sites. Human telomeric DNA, which ranges from 5 to 12 kb,, consists of 5’-TTAGG-3’ repeat sequences. They are bound by Shelterin complex comprising of TRF1 (telomere repeat-binding factor 1), TRF2 (telomere repeat-binding factor 2), POT1 (protection of telomeres 1), TPP1 (telomere-binding protein POT1-interacting protein 1), TIN2 (TRF1-interacting nuclear protein 2) and RAP1 (Repressor/activator protein 1) [1]. Due to the end-replication problem, the telomeres of somatic cells shorten by 50 to 150 bp with each cell division until a DNA damage response (DDR) is elicited, such as G1 phase arrest and cellular senescence [2]. The activation of the telomere maintenance mechanism (TMM) enables tumor cells to achieve replicative immortality and evade cellular senescence. While most cancer cells maintain telomere length though telomerase activation, approximately 5–10% of cancers engage the alternative lengthening of telomeres (ALT) pathway for telomere maintenance [3]. The prevalence of ALT is notably greater in certain cancer types, including sarcomas, isocitrate dehydrogenase-mutant astrocytoma (WHO grade II–IV), pancreatic neuroendocrine tumors, neuroblastoma and chromophobe hepatocellular carcinomas [4]. ALT cells are characterized by an increased incidence of extrachromosomal circular telomeric DNA (C-circles and T-circles), an elevated frequency of telomeric sister chromatid exchanges (T-SCEs), the formation of ALT-associated PML (promyelocytic leukemia) bodies (APBs, which are nuclear structures that assemble specifically in ALT-positive cells and are important for telomeric DNA synthesis), and frequently harbor inactivating mutations in chromatin remodeling proteins (such as ATRX and DAXX) or DNA damage repair factors (such as SMARCAL1 and SLX4IP) [5–7]. The ALT pathway has long been speculated to be a homologous recombination-based process that depends on break-induced replication (BIR)-mediated DNA synthesis to produce novel TTAGGG repeats [8]. Although recent studies have identified DNA synthesis pathways of ALT telomeres, such as RAD51-dependent and RAD51-independent, RAD52-dependent and RAD52-independent pathways [9–11], it is still unclear what conditions trigger or determine the selection of those telomere synthesis pathways. Telomeres pose a challenge to DNA replication because of their repetitive nature and potential to form secondary structures such as telomere loops (T-loops), DNA/RNA hybrids (D-loops) and G-quadruplexes (G4s). This replication challenge is further exacerbated in ALT tumor cells because ALT telomeres exhibit increased replication stress that functionally perpetuates telomere extension. Although the mechanism of ALT activation in cancer cells remains poorly understood, cumulative evidence suggests that replication stress at telomeres may trigger ALT [12, 13]. Supporting this, the ATRX/DAXX complex is commonly mutated in ALT cell lines, leading to nucleosome assembly defects that likely contribute to chromatin remodeling and increased replication stress [13, 14]. Depletion of the histone chaperone ASF1 results in increased replication stress at telomeres and the induction of ALT phenotypes [15]. It’s worth noting that depletion of some replication fork restart/reversal factors, such as SMARCAL1, FANCD2 and FANCM, increases ALT activity but also inhibits ALT cell proliferation [16–18], indicating that the replication stress at telomeres is tightly regulated. The increased stress at telomeres causes double-strand breaks (DSBs), which may favor BIR, resulting in elongated telomeres and establishing ALT [8, 19]. In this mechanism, a resected 3’-overhang invades a homologous dsDNA sequence, forming a displacement loop and initiating DNA synthesis [20]. Since ALT-mediated telomere elongation employs homologous recombination-based events, it suggests an important role for DNA helicases in the dissolution of recombination intermediates. Indeed, two members of the RecQ helicase family, BLM and WRN (the proteins mutated in Bloom’s syndrome and Werner’s syndrome), are shown to play important roles in telomere elongation during ALT processes. Both WRN and BLM localize to APBs and bind to ALT telomeric DNA [21, 22]. WRN is required for efficient lagging strand synthesis of telomeres, likely because WRN unwinds G-quadruplex structures during telomere replication [23]. WRN has been shown to prevent telomere loss in VA13/WI38 and U2OS ALT cells by suppressing aberrant recombination within telomeres [24]. However, activation of ALT phenotypes is also observed in WRN-deficient cells, suggesting that WRN may be dispensable for ALT [25]. In contrast to WRN, BLM appears to be more closely associated with the ALT mechanism. BLM colocalizes with telomeres in ALT human cells but not in telomerase-positive immortal cell lines or primary cells [22]. Depletion of BLM leads to telomere shortening in ALT cells but does not affect telomere length in cells immortalized by telomerase [26]. Furthermore, BLM overexpression results in ALT-specific accumulation of telomeric DNA [22]. BLM functions to dissolute BIR recombination intermediates as a component of the BTR complex (composed of BLM helicase, topoisomerase TOP3α, RMI1 and RMI2) [22, 27], which is critical for triggering ALT since tethering the BTR complex to telomeres is sufficient to induce ALT phenotypes in non-ALT cells [28]. Additionally,, BLM plays additional roles in recombination to promote the ALT pathway by recruiting endonucleases such as DNA2 or EXO1 for 5’ end resection and cooperating with POLD3 in branch migration and template copying of the invading strand [29–31]. Although BLM is known to be critical for ALT, there are still many questions about its exact function in ALT. For instance, although BLM is required for ALT telomere maintenance and genomic stability, in certain contexts, such as FANCM-depleted, SLX4/SLX4IP-depleted and EXD2-depleted cells, BLM may cause toxicity by eliciting hyperactive ALT and associated telomere dysfunction [7, 32, 33]. Is the function of BLM in regulating ALT is associated with different genetic backgrounds? This review will discuss the BLM functions in ALT, including processing recombination intermediates during BIR or enhancing DSB end resection, and explores emerging therapeutic strategies targeting ALT-positive cancers.

## BLM: structure, binding partners, and function in DNA metabolism

### The domains and expression of BLM helicase

**Fig. 1:**
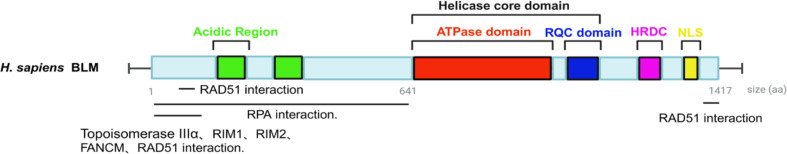
Schematic representation of the structural domains of BLM from *Homo sapiens.* Abbreviations: NLS, nuclear localization sequence; HRDC, helicase and RNase D-like C-terminal domain; RQC, RecQ C-terminal domain. The figure shows interacting proteins and their direct binding sites in the regulation of ALT

The BLM protein consists of 1,417 amino acids and shares three highly conserved protein domains with the RecQ helicase DNA subfamily: the core helicase domain (which contains an ATP binding site and an Asp-Glu-x-His (DExH) sequence), the RecQ C-terminal (RQC) domain, and the helicase and RNase D-like C-terminal (HRDC) domain (Fig. 1) [34]. The helicase activity is required for unwinding a wide variety of DNA substrates, many of which resemble DNA repair intermediates, such as DNA G4s, R-loops, D-loops, Holliday junctions (HJs) and stalled replication forks [35]. This activity also indicates that BLM functions in correcting the genomic instability characteristic of Bloom syndrome (BS)cells [36]. The minimal helicase functional unit of BLM also includes an RQC domain, which specifically mediates binding to G4 DNA and other DNA structures [37]. The helicase activity, along with interactions with TOP3α and RMI1 to form the BTR complex, is essential for possibly all BLM functions (Fig. 1).

The most well-studied mutant of BLM is K695A, which is completely defective in both ATPase activity and helicase unwinding activity [38]. The HRDC domain in BLM plays a role in recruiting it to specific DNA lesions [39], and this region is thought to contribute to BLM’s conformational change [40]. A nuclear localization signal (NLS) has been identified in the carboxyl-terminal region of BLM [34]. BLM functions as a single-stranded (ss) DNA translocase by interacting physically and functionally with ssDNA-binding (SSB) proteins, such as replication protein A (RPA) and RAD51 [41–43], thus highlighting the multifaceted genome maintenance function of BLM.

The expression of BLM is regulated by the cell cycle. BLM protein accumulates at high levels during S phase, persists in G2/M, and is undetectable in G1, suggesting rapid degradation during mitosis [42, 44]. This regulation is likely tied to BLM’s function in DNA replication in the S phase and homologous recombination in the G2 phase. Notably, during S phase, BLM colocalizes with RPA at replication foci and restarts stalled replication forks [45]. Cdc5-mediated hyperphosphorylation of BLM may reduce its DNA unwinding activity during mitosis [46]. In response to DNA damage, the localization and expression regulation of BLM are altered. For example, treatment with hydroxyurea (HU) induces the relocalization of BLM to RAD51 and p53 foci at sites of stalled DNA replication forks to inhibit homologous recombination and help maintain genomic integrity [47]. Furthermore, HU treatment leads to a significant increase in BRCA1 and BLM colocalization, which appears to be specific to cells in the S and G2 phases [48].

### BLM’s interaction proteins and functions

The execution of certain BLM functions relies on its interactions with other proteins. BLM forms a BTR complex and plays a key role in the resolution of intertwined DNA structures during DNA replication and DNA damage repair [49]. BLM can interact with RPA to unwind DNA duplexes during replication, recombination, or repair [50]. In addition, BLM has been shown to interact with proteins critical for proper DSB repair, such as BRCA1, MLH1, FANCJ/M, EXO1, FEN1 and the MRN complex (MRE11-RAD50-NBS1); the topoisomerases TOP1 and TOP2α; the DNA damage response proteins p53, 53BP1, and H2AX; the telomere-binding proteins TRF1, TFR2, and POT1; the helicase-like proteins PICH, polymerase Polη and Polδ; and others [51, 52].

#### DNA replication

BLM helicase is involved in several replication-associated events of DNA replication, including Okazaki fragment processing, DNA strand elongation, and the resolution of replicative stress. BLM interacts with FEN1, a 5’-flap endonuclease/5’-3’ exonuclease, indicating its role in Okazaki fragment maturation [53]. BLM also participates in extension of the leading strand by interacting with p12, the smallest subunit of human Polδ, suggesting that BLM might be recruited to replication sites [54]. The physical interaction between BLM and 53BP1 leads to Chk1-mediated S-phase arrest, suggesting a potential role of BLM in the DNA replication checkpoint [55]. The helicase activity of BLM is thought to play important roles in replication fork restart. Replicative stress can be induced by various factors, including protein‒DNA complexes, RNA: DNA hybrids and accumulated atypical DNA structures such as quadruplexes, hairpins or HJs. BLM is essential for stabilizing stalled replication forks and promoting fork progression, mainly due to its ability to unwind unusual DNA secondary structures and prevent hyperrecombination [49, 56]. Fork regression, involving the repair of parental strands and the annealing of nascent daughter strands to form a “chicken foot” or HJ intermediate, is believed to be the initial event in response to replication blockage. BLM has been shown to function in “reverse” branch migration to restart the replication fork though its strand-annealing activity [57]. BLM may also prevent the formation of UFBs (ultrafine anaphase bridges), which correspond to either incompletely replicated DNA sequences or unwound structures and must be resolved before the end of cell division to ensure sister-chromatid disjunction. Most UFBs are of centromeric origin, whereas some UFBs induced by replication stress originate from common fragile sites and telomeres or ribosomal DNA repeats [58, 59]. In cooperation with the primary UFB-binding factor PICH (PLK1-interacting checkpoint helicase), BLM is recruited to UFBs to resolve toxic DNA catenanes [60].

#### DNA repair

Given that BLM interacts with proteins involved in DNA repair and prefers binding to DNA substrates that resemble DNA repair intermediates, it suggests that BLM plays a role in DNA repair pathways. BLM is thought to primarily affect DSB repair. DSBs are critical DNA damage events that cause mutations and genome instability, eventually leading to cell death or tumorigenesis. In mammalian cells, DSBs are repaired mainly by non-homologous end-joining (NHEJ, also known as c-NHEJ), alternative non-homologous end joining (a-NHEJ) and homologous recombination (HR). Emerging evidence supports a role for BLM in HR. BLM physically interacts with HR proteins, such as RAD51 and BRCA1 [47, 48]. DSB end resection is one of the earliest steps of recombinational DNA repair, and is mediated by 3’-5’ helicase and 5’-3’ nuclease activity. BLM is an essential component of both the DNA2-dependent and EXO1-dependent pathways for long-range resection [29]. BLM can also selectively bind Holliday junctions and promote ATP-dependent branch migration [61]. However, in some cases, BLM shows anti-recombination activity. It can disrupt the RAD51-ssDNA nucleoprotein filament by dislodging the human RAD51 protein from ssDNA, which disrupts the D-loop formation and HR initiation [62]. In cells lacking BRCA1 or BRCA2, ablation of BLM rescues genomic integrity and defective HR by allowing the accumulation of RAD51 at resected DSBs, resulting in cell survival in the presence of DNA damage [63]. Therefore, BLM displays both pro-and anti-recombinogenic activities, each of which contributes to the maintenance of genomic integrity.

#### Telomere maintenance

BLM mutant cells are characterized by an excess of various chromatid lesions, including hyper-recombination and telomere associations, which is defined as a phenotype in cells with defective telomere maintenance [41], suggesting a role for BLM in telomere maintenance. Telomeres are especially susceptible to replication stress because of the enrichment of secondary structures such as G-quadruplexes, D-loops, and T-loops [64]. It is essential to remove G-quadruplexes and unwind T-loops to prevent fork stalling and telomere loss during replication, which may partly explain the importance of RecQ helicases in telomere replication. In addition, BLM interacts with shelterin proteins, such as TRF1, TRF2 and POT1 to regulate the unwinding of telomeric D-loops, suggesting a function for BLM in cells that employ recombination-mediated telomeric DNA synthesis [22, 65, 66]. Telomeres and chromosomal fragile sites (CFSs) are specific loci that are prone to breakage, probably due to their difficult-to-replicate characteristics. BLM helicase cooperates with the MUS81-EME1 resolvase complex to prevent uncontrolled chromosome breakage in CFSs in normal cells [67]. In BLM-deficient cells, G4 accumulation is observed, causing telomere fragility [68], which indicates a function of BLM in resolving telomere and whole genome replication problems. However, in some studies, primary cells derived from BS patients demonstrate comparable telomere length to age-matched controls, indicating that BLM may not be a major regulatory factor in maintaining telomere length [41]. Rather than unwinding G4 or preventing telomere fragility, BLM exhibits more complicated functions in ALT cells, either by promoting the correct replication of telomeres or dissociating HR intermediates. The detailed mechanism of BLM in ALT will be further discussed below.

### The role of BLM in ALT activation

Owing to the dysregulated telomeric chromatin status, the interspersion of telomere variant repeats, the elevated levels of TERRA (Telomeric repeat-containing RNA), and abundant telomeric R-loops, the replication of ALT telomeres is more stressful, resulting in the accumulation of higher levels of telomeric DNA damage [69–71]. It has been proposed that elevated telomeric replication stress promotes break-induced telomere synthesis at ALT telomeres. The balance between extensive DNA damage and homology-directed repair is precisely regulated to activate or maintain ALT mechanisms, and BLM is one of the known drivers of ALT. In terms of regulating replication stress, BLM’s helicase activity is required to reduce replication stress and promote ALT-associated phenotypes by unwinding G-quadruplexes and duplex DNA [22]. Loss of FANCM, a DNA translocase that interacts with the BTR complex and suppresses telomeric R-loops, increases replication stress at telomeres and enhances ALT features [32]. BLM is likely to contribute to end resection in FANCM-depleted ALT cells, which leads to hyper-activation of ALT and toxicity in cells [32]. However, the helicase activity of BLM has also been shown to be required for the generation of replication stress and BIR at ALT telomere [72]. On the other hand, BLM might promote homology-directed repair by both enhancing end resection or processing recombination intermediates. In a most recent report, BLM is also shown to be responsible for assembling the ALT telomere DNA damage response through its helicase-dependent genesis of 5’-ssDNA flaps on lagging strand telomeres, suggesting that the helicase activity of BLM at lagging strand telomeres would be the initiating event of ALT [73].

### BLM drives ALT by promoting APB formation

**Fig. 2:**
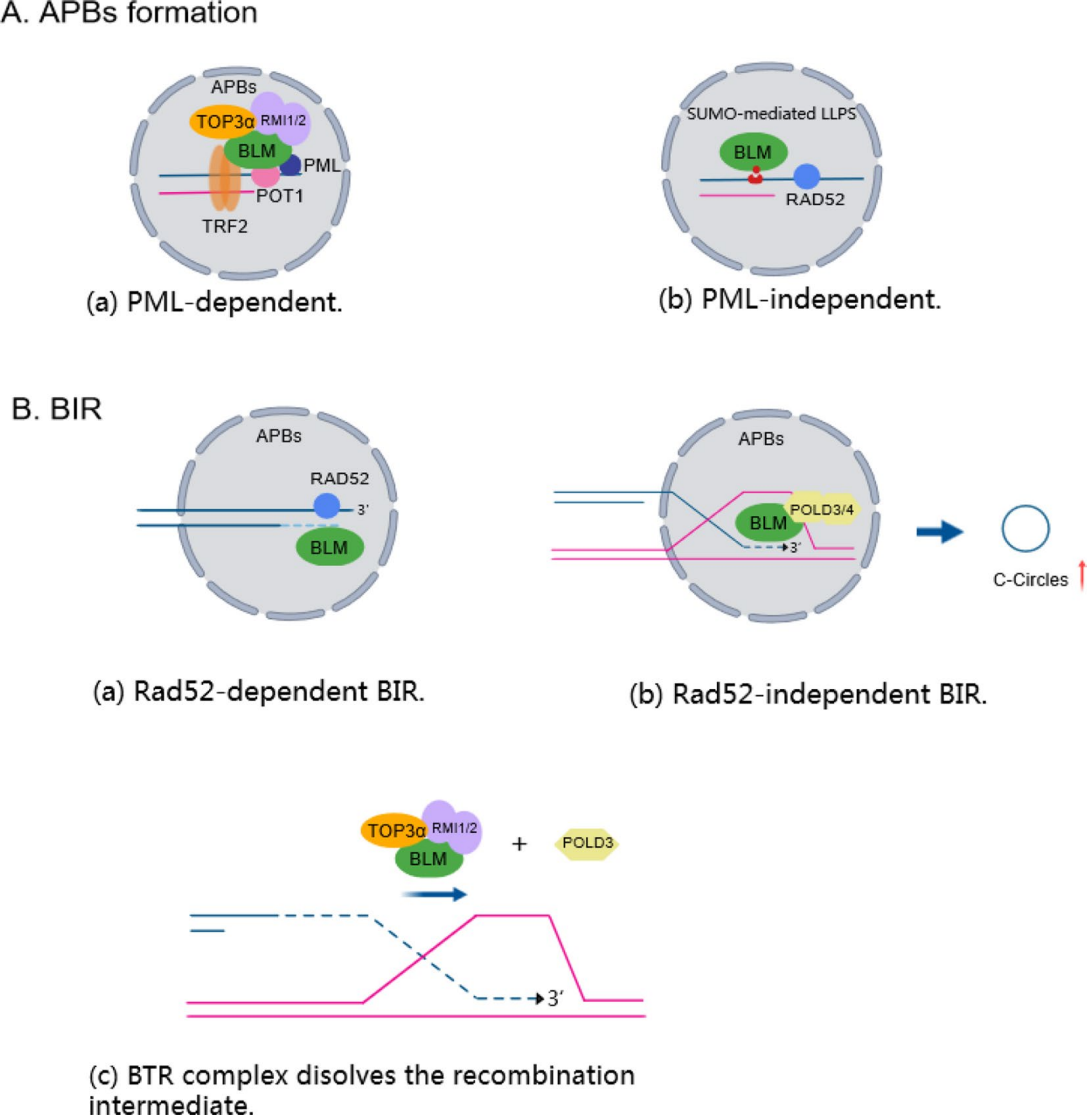
BLM drives ALT by promoting APB formation and BIR. **A**. The BTR complex and TRF2 or POT1 colocalize on telomeres and promote APB formation in a PML-dependent manner (**a**). The SUMO-SIM interactions of telomeres with BLM and RAD52 can induce the formation of nuclear structures via LLPS (**b**). **B**. BLM is required for the initiation of RAD52-dependent BIR through 5’ to 3’ end resection (**a**). In RAD52-independent BIR, BLM is also responsible for telomere synthesis and C-circle formation (**b**). The BTR complex is essential for the dissolution of recombination intermediates, resulting in POLD3-dependent ALT telomere extension

Emerging evidence shows that telomere clustering and BLM are required for the onset of the ALT pathway. APBs are unique nuclear structures that are present specifically in ALT cells, and are considered as a “recombinogenic microenvironment” and bring clustering telomeres and DNA repair proteins to promote ALT [74]. BLM and TRF2 or POT1 colocalize in APBs and stimulate BLM to unwind telomeric forked duplexes and D-loop structures, thereby promoting recombination-driven amplification of telomeres in ALT cells [22, 26, 65, 66]. However, the interaction between BLM and TRF1 inhibits BLM unwinding of telomeric substrates, possibly because TRF1 and TRF2 function to coordinate BLM activity in cells using ALT [66]. (Fig. 2, A). Intriguingly, the N-terminus of BLM was shown to be required for its telomere binding during the S/G2 phase [75], and the colocalization of BLM with TRF2 and PML reached a maximum during the late S, G2 and M phases, which is consistent with the reported enrichment of APBs during G2/M when ALT is initiated [66]. Depletion of BLM with siRNAs drastically abolishes APB and ALT telomere synthesis in G2 [9, 15], whereas overexpression of the BLM helicase not only results in the localization at APBs in the presence of telomere clustering induced by engineering poly(SUMO)/poly(SIM) scaffolds but also triggers hallmarks of the ALT pathway in ALT cells [76]. By tethering PML-IV (a splicing variant of PML, which is the only PML variant that restors telomere clustering and telomere synthesis in PML knockout cells) to telomeres, BLM was observed to induce replication stress in APBs, which is essential for ALT telomere synthesis [72].

Moreover, the SUMO-SIM interactions of telomeres with BLM, RAD52 and RAD51AP1 can induce the formation of nuclear structures with features of liquid‒liquid phase separation (LLPS) and reminiscent APBs formation in a PML-independent manner [77](Fig. 2, A).

### BLM is required for maintaining telomere structure and length in ALT cells

BLM drives ALT not only by promoting APB formation and telomere clustering but also by processing BIR intermediates and facilitating mitotic DNA synthesis (MiDAS). BIR is known as homology-directed DNA synthesis, which has been proceeded by strand invasion followed by the migration of a D‐loop intermediate [78, 79]. The D‐loop intermediates can be processed either by resolution (catalyzed by structure‐specific endonucleases) or by dissolution (catalyzed by the RecQ helicase) [80, 81]. Disruption of BLM causes telomere length attrition only in cells using ALT, which is likely due to the helicase activity of BLM functioning at the stalled replication forks within the ALT telomere DNA [26]. In cells harboring PML-IV-induced APBs, loss of BLM helicase activity leads to a dramatic increase in telomeric anaphase bridges in mitotic cells, suggesting that BLM promotes ALT telomere extension by resolving BIR intermediates via its helicase activity [72]. In addition to its helicase activity, the BTR complex is essential for the dissolution of recombination intermediates, resulting in POLD3‐dependent ALT telomere extension [22, 31]. Furthermore, PML is responsible for the localization of the BTR complex to ALT telomere ends to trigger ALT phenotypes and telomere synthesis, which attests to the importance of BTR in telomere synthesis and triggering ALT [28](Fig. 2, B).

MiDAS is known to proceed via a RAD52-dependent BIR-like process, which has been proven to be strongly related to the activation of ALT [76, 82]. The helicase activity of the BLM protein, possibly involved in 5’-to-3’ long-range resection processes, is required for telomere replication and repair during the mitotic phase [76]. Recent studies have shown that even in the absence of PML and APBs, BLM is enriched at ALT telomeres by the SUMOylating and SUMO-SIM interaction, which can induce phase-separated condensates that contain both telomeres and DNA repair proteins, leading to telomere DNA synthesis [77]. Moreover, in RAD52-knockout ALT cells, C-circle formation and telomere synthesis are dependent on BLM and the BIR proteins POLD3 and POLD4 [83], suggesting a new role for BLM that does not depend on end resection in BIR pathway. As a conclusion, BLM can promote ALT telomere synthesis in various ways, such as dissolving HR intermediates, forming BTR complexes, and promoting liquid‒liquid phase separation.

### The BTR complex is required for the generation of cECTRs in ALT cells

The presence of circular extrachromosomal telomeric repeats (cECTRs, including partially single-stranded telomeric CCCTAA repeats, known as C-circles, and intratelomeric duplex DNA repeats, known as T-circles) has long been associated with ALT development and is considered as a diagnostic marker for ALT tumors [84]. Although the origin and function of cECTRs in ALT remain elusive [85, 86], they are currently thought to play two main opposing roles in telomere maintenance. cECTRs have been suggested to be associated with telomere loss due to telomere-trimming events and HR-mediated telomere maintenance. On the other hand, they can also serve as templates for rolling-circle-like amplification and promote telomere elongation in ALT cells [87, 88]. Depletion of BRCA1 and BLM decreased the number of C-circles in all ALT cell lines [89]. In addition, loss of the BTR complex also results in a significant reduction in cECTRs, suggesting that BLM may be required for the formation of cECTRs in ALT mechanisms [28, 31]. Depletion of the histone chaperones ASF1a and ASF1b in human cells induces all ALT-like phenotypes, including BLM-dependent cECTRs [15]. Similarly, overexpression of the telomere-binding protein TZAP induces ALT-like activity and promotes T-circle and C-circle excision, leading to telomere trimming in ATRX/DAXX deficient cells, and the BTR complex is required for this TZAP-induced generation of cECTRs [90]. Since ASF1 functions primarily in replication coupled chromatin assembly and its depletion affects replication fork progression in S-phase; and since loading of TZAP onto telomeres in ALT cells also causes telomeric replication stress, these findings suggest a role for BLM or the BTR complex in alleviating replication stress by interaction with fork restart/reversal factors, such as FANCD2 and FANCM [18, 32]. Recent research has demonstrated that internal telomeric cECTRs called I-loops, which are induced by single-stranded damage at normal telomeres and identified as the majority of telomeric structures in ALT tumor cells. These I-loops could be substrates for some indicated helicases, such as BLM, to generate telomeric circles induced by chronic telomere damage in ALT cells [91]. However, this conclusion requires further validation.

## The regulation of BLM in the ALT mechanism

**Fig. 3:**
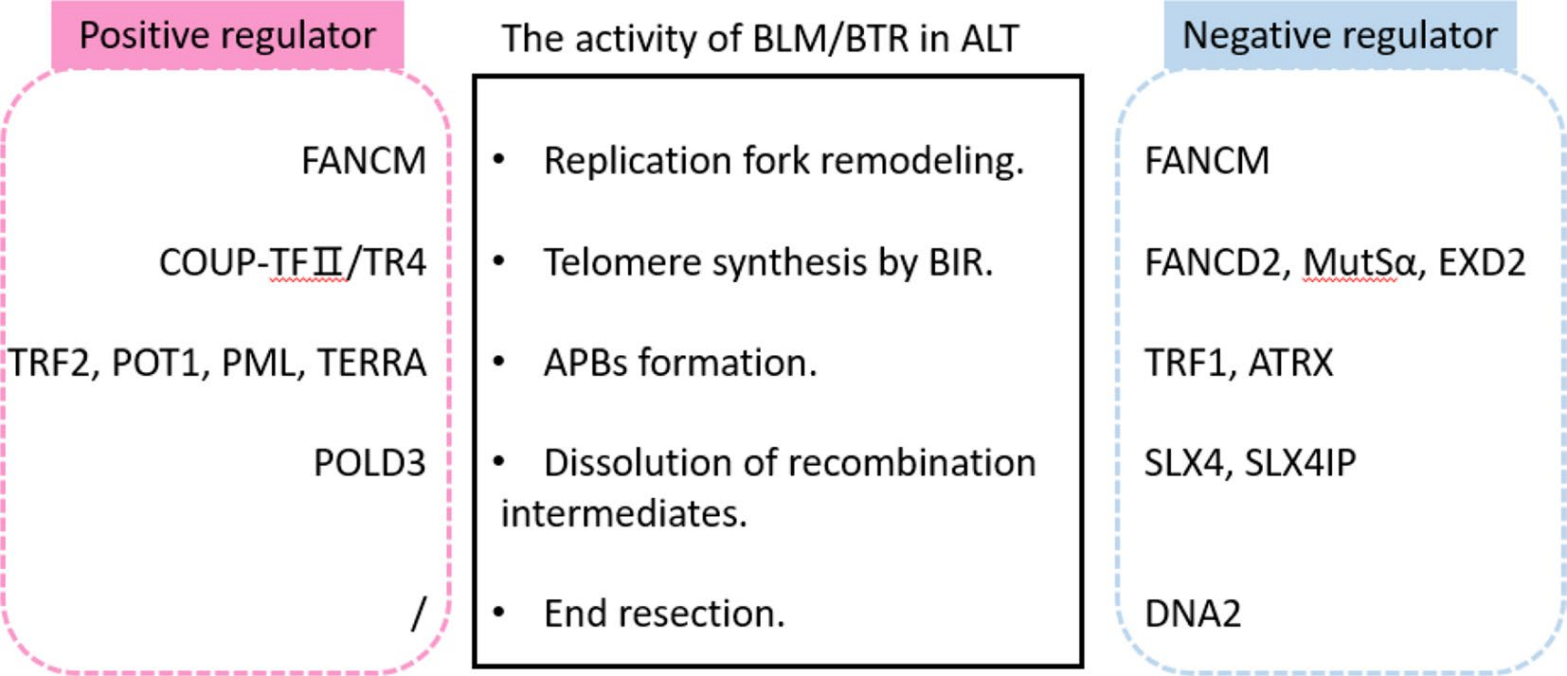
Summary of the positive and negative regulators that modulate the activity of BLM or the BTR complex in ALT

ALT is believed to proceed in the yeast *Saccharomyces cerevisiae* via two separate pathways, termed type I and type II ALT [92]. Type I ALT in yeast is RAD51-dependent and relies on 5’-3’ resection to initiate inter‐telomeric recombination and telomere synthesis, while type II ALT in yeast is RAD51‐independent and is proposed to involve a break-induced replication process [93, 94]. Recent research on human cancer cell lines has indicated the conservation between these two distinct ALT mechanisms in yeast and the ALT pathway in human cancers [8, 31, 93, 95]. While both ALT pathways rely on BLM and the PCNA-RFC-Polδ replisome [9], the RAD51-dependent pathway is associated with inter-telomere recombination during the S/G2 phases, and BLM coordinates with RAD51 for the initiation of telomere synthesis [31, 96]. The RAD51-independent ALT-like mechanism in human cancer is considered the dominant mechanism of telomere lengthening during the G2/M phase, which facilitates RAD52-dependent BIR for telomere synthesis and requires BLM for resolving BIR intermediates, recruiting RAD52 and other DDR proteins to APBs, and generating replication stress [72]. This pathway is also compensated by a RAD52-independent but BLM-dependent pathway to produce C-circles [9, 76]. Since the function of BLM in ALT pathways is complex and precisely regulated, here, we attempt to sort out the regulators of BLM in the ALT mechanism according to the latest studies (Fig 3).

### Factors that promoted BLM activity in ALT

Besides the shelterin complex and PML, which can recruit BLM to APBs, recent studies have emphasized the FANCM-BTR complex as a key regulator of ALT homeostasis [17], which may have evolved to process both G4s by BLM [97] and R-loops by FANCM [32]. FANCM binds to the BTR complex via its MM2 domains, and the FANCM-BTR is essential for replication fork remodeling of ALT telomeres, thereby maintaining telomere integrity [17]. Disrupting the FANCM-BTR exacerbates DSBs and ultimately results in the loss of ALT cell viability [32]. Depletion of COUP-TFII/TR4 resulted in a significant decrease in BLM signals at telomeres, suggesting that COUP-TFII/TR4 might induce different DNA repair pathways in ALT besides the FANCM pathway [98]. BLM was also found to interact with TERRA [99], and the depletion of TERRA resulted in the reduction of BLM recruitment to ALT telomeres and telomere clustering, indicating that TERRA could serve as a scaffold to recruit BLM and other ALT-associated proteins, such as RPA, to promote APB formation [100].

### Factors that restrict BLM activity in ALT

Although overexpression of BLM recapitulates important steps for ALT activation [76], BLM activity seems to contribute to the hyper-ALT phenotypes, leading to genomic toxicity and ALT cell death. Thus, many proteins have been reported to interact with BLM to limit its activity in ALT. Similar to BS, genomic instability syndrome Fanconi anemia (FA) is caused by mutations in FA proteins that participate in DNA repair processes and replication pathways [101]. Twenty-two distinct FA genes have been identified to date. In response to DNA damage, eight FA proteins (A, B, C, E, F, G, L, and M) form the FA “core” complex that recruits and monoubiquitylates two downstream FA proteins, FANCD2 and FANCI. The mono-ubiquitylation of FANCI-D2 complex then recruits and activates other DNA damage response proteins, which remove interstrand crosslinking (ICL) sites and induce HR to repair DNA damage [101]. Several FA proteins are directly associated with ALT telomere replication. For instance, FANCD2 localizes to APBs, and depletion of FANCD2 leads to hyperactivation of ALT-associated phenotypes, including increases in telomere length, APB size, ECTR number, telomere dysfunction induced foci (TIFs) and fragile telomeres in ALT cells. Moreover, the co-depletion of BLM suppresses these hyper-ALT phenotypes. Mechanically, the monoubiquitylation of FANCD2 plays an antagonistic role in restraining BLM from over-resection of stalled forks in ALT cells, thus inhibiting telomere replication and recombination [18]. In addition, depletion of FANCD2 increases the signals of BLM at telomeres [98], indicating FANCD2 is a negative regulator of the BLM-dependent telomeric DNA synthesis pathway in ALT cells.

FANCM also restricts uncontrolled BLM [32]. Mammalian FANCM homologs mediate branch migration, replication fork reversal and 3’-5’ DNA helicase activity [102]. In ALT cells, depletion of FANCM induces robust telomere replication stress and damage, elevated APBs and ECTRs, and the accumulation of BLM and BRCA1 at ALT telomeres [32, 103]. In addition, FANCM directly displaces BLM from telomeres. Although both FANCD2 and FANCM have been shown to suppress BLM toxicity in ALT cells, this is unlikely due to the general role of the FA pathway since the expression of the FANCM MM1 domain mutant in ALT cells is unable to recruit the FA complex to chromatin and suppress all ALT-associated features [17].

SLX4IP (SLX4 interacting factor) and SLX4 counter BLM-mediated telomere clustering to coordinate the resolution and dissolution of recombination intermediates in the ALT mechanism [7, 31]. The telomere replication intermediates, such as D-loops and HJs are substrates for both the SLX4-nuclease complex and the BLM helicase. The helicase activity of BLM is able to suppress the nuclease activity of SLX4 in processing telomeric D-loops and HJs in vitro [104]. It was demonstrated that the ALT-mediated telomere synthesis, which is followed by BTR complex-modulated telomeric dissolution is counteracted by the SLX4-SLX1-ERCC4 complex, which prematurely resolves the recombination intermediate after telomere strand invasion [31]. SLX4IP binds to both BLM and SLX4 and counterbalances the dissolution activity of BLM to ensure the appropriate processing of ALT telomeres and prevent telomere breakage [7].

Mismatch repair (MMR) is initiated by heterodimers MSH2/MSH6 (MutSα) or MSH2/MSH3 (MutSβ), where MSH3 and MSH6 compete for binding to MSH2 [105]. The MutSα complex is proved to interact with the BLM helicase and stimulate its ability to process Holliday junctions in vitro [106]. However, a recent study has shown that the MutSα complex occupies telomeres specifically in ALT cancer cells and restricts telomere extension, in part by counteracting the recombination function of the BTR complex [70]. In addition, disrupted BLM in MutSα-deficient ALT cells increases the discrete banding of telomere fragments and exhibits a defect in the ability to form colonies, suggesting that ALT cancer cells rely on BLM to maintain telomere integrity and cellular survival when faced with the hyperextension of telomeres caused by MutSα deficiency [70]. BLM is also responsible for the elevated levels of C-circles in MSH3-depleted U2OS cells, given that MSH3 and MSH6 compete for MSH2 to form heterodimers, which suggests a complicated role of the interaction between BLM and MutSα in ALT cells [107].

In a recent study, a 3’-5’ exonuclease, EXD2, plays a critical role in determining repair pathway choice in the ALT mechanism. It is found that EXD2 acts within the same pathway as SLX4 to suppress RAD52-independent BIR in ALT cells, which is mediated by BLM. EXD2-deficient cells engage BLM-dependent conservative mitotic telomere synthesis, and co-depletion of EXD2 with BLM confers synthetic lethality in ALT cells, suggesting that EXD2 functions on ALT telomeres via a parallel pathway to BLM [108]. BLM is also required for the initiation of telomere clustering and telomere synthesis through long-range 5’ to 3’ end resection (with DNA2 and EXO1) [29, 109, 110]. In a recent report, BLM recognizes single-stranded nicks of C-rich sequence on lagging strand telomeres and promotes 5’-flap formation, supporting a role of BLM in providing extensive replication stress-associated damage response and therefore initiate HR in ALT [73]. However, the DNA2 endonuclease cleavage this long 5’-flaps to limit ALT [73].

## Future directions

Cells derived from Bloom syndrome patients exhibit multiple indications of genome instability such as increased chromosomal aberrations and elevated sister chromatid exchanges (SCEs), indicating a genome guardian function. By promoting branch migration, the helicase activity and interaction with TOP3α and RMI1 to form the BTR complex are important for dHJ dissolution, which results in non-crossover DNA repair products [111]. BLM also maintains genome stability via other mechanisms, such as mediating ultrafine DNA bridges in anaphase (with PICH) [112], and restarting stalled replication forks (with RPA) [45].

There is no doubt that BLM is critical for regulating telomere hemostasis in ALT; however, how it functions in ALT still requires future investigation. In a research that focuses on identifying protein partners of BLM in ALT cells, telomerase-associated protein 1 (TEP1), heat shock protein 90 (HSP90), and topoisomerase II alpha (TOP2α) were found to bind directly to BLM and TRF2 only in ALT cells [26]. In vitro helicase assays show that TOP2α slightly slows the kinetics of BLM unwinding of telomeric substrates. In contrast, HSP90 inhibits BLM unwinding of both telomeric and non-telomeric substrates [26]. However, future studies still need to determine the precise role of each protein in ALT pathways and how these proteins interact with BLM to carry out telomere elongation. Besides, a component of the splicing factor 3b protein complex,, SF3B2 was also identified as a BLM interaction protein in ALT cells; however, there have been no reports of its role in ALT or its interaction with BLM thus far [26]. It is also worth noting that ATRX, whose deficiency is a well-known driver of ALT, diminishes BLM localization to ALT telomeres along with reductions of APBs. However, the dependence of BLM on ATRX loss to direct ALT remains undefined [73].

The role of BLM in dHJ dissolution is well established, but the extent to which BLM functions in regulating HR is less clear. It was revealed that BARD1 recruits SLX4 and MUS81 to resolve DNA intermediates left unprocessed by BLM, given the possibility that the BTR complex dissolve most replication intermediates prior to cell division. However, BLM may not deal with all joint molecules before anaphase. Then BRCA1-BARD1 recruited SLX4 at the G2/M to cleavage the leftover replication intermediates and produce SCEs [113].

As mentioned above, the ALT mechanism relies on a delicate balance between high levels of DNA damage at telomeres and recombination-dependent repair. Therefore, altering the level of DNA damage and disrupting the repair system may be promising ways to target tumors that dependent on ALT. Indeed, an inhibitor of the ATR kinase (VE-821) selectively kills ALT cells in vitro, which may be attributed to increased replication stress and diminished DDR at telomeres [114]. However, more findings support the idea that the tumor-suppressing effects of ATR inhibitors are independent of the ALT mechanism, indicating that other targets need to be explored [115, 116]. In addition, strategies that inhibit telomeric homologous recombination also show potential for targeting ALT cancer cells. For example, the cisplatin derivative Tetra-Pt(bpy) can specifically kill ALT tumor cells by stabilizing G4s, thereby inhibiting the strand invasion/annealing step of homologous recombination [117]. The tyrosine kinase inhibitor ponatinib exacerbates replicative stress and limits telomere synthesis, resulting in highly effective killing of ALT cells [118]. Since BLM plays an important role in regulating both replication stress and telomere synthesis in ALT, which has revealed its vulnerabilities in ALT tumor therapy. However, there are few reports on therapeutic target of BLM helicase, among which ML216 is the first and only commercial small molecule inhibitor that competitively inhibits the DNA-binding activity of BLM [119]. It has been shown that ML216 combined with Olaparib, exerts a synergistic radio sensitization effect on Olaparib-resistant non-small cell lung cancer cells by inhibiting HR repair, and ultimately inducing apoptosis [120]. However, there are no reports of its effect on ALT cells yet. The dissection of BLM’s function in ALT and the mechanism of BLM inhibitors in ALT will guide future strategies to eliminate ALT-reliant cancers.

## Data Availability

Not applicable.

## Abbreviations

ALT: Alternative lengthening of telomeres
APBs: ALT-associated promyelocytic leukemia bodies
a-NHEJ: Alternative non-homologous end joining
BIR: Break-induced replication
BS: Bloom syndrome
BTR complex: A complex formed by BLM helicase, topoisomerase TOP3α, RMI1 and RMI2
cECTRs: Circular extrachromosomal telomeric repeats
CFSs: Chromosomal fragile sites
c-NHEJ: Classic non-homologous end-joining
DDR: DNA damage response
D-loops: DNA/RNA hybrids
DSBs: Double-strand breaks
dsDNA: Double-stranded DNA
FA: Fanconi anemia
G4: DNA G-quadruplex
LLPS: Liquid‒liquid phase separation
HJ: Holliday junctions
HR: Homologous recombination
HU: Hydroxyurea
ICL: Interstrand crosslinking
MiDAS: Mitotic DNA synthesis
MMR: Mismatch repair
PML: Promyelocytic leukemia
ssDNA: Single-stranded DNA
SCEs: Sister chromatid exchanges
TERRA: Telomeric repeat-containing RNA
TIFs: Telomere dysfunction induced foci
TMM: Telomere maintenance mechanism
T-SCEs: Telomeric sister chromatid exchanges
T-loops: Telomere loops
UFBs: Ultrafine anaphase bridges
